# Attachment Quality and Psychopathological Symptoms in Clinically Referred Adolescents: The Mediating Role of Early Maladaptive Schema

**DOI:** 10.1007/s10826-012-9589-x

**Published:** 2012-05-04

**Authors:** Jeffrey Roelofs, Linda Onckels, Peter Muris

**Affiliations:** RIAGG Maastricht, Child and Youth Care and Clinical Psychology Science, Faculty of Psychology and Neuroscience, Maastricht University, P.O. Box 616, 6200 MD Maastricht, The Netherlands

**Keywords:** Attachment insecurity, Early maladaptive schemas, Psychopathological symptoms, Clinically referred adolescents

## Abstract

This study investigated relationships between attachment insecurity, maladaptive cognitive schemas, and various types of psychopathological symptoms in a sample of clinically referred adolescents (*N* = 82). A mediation model was tested in which maladaptive schemas operated as mediators in the relations between indices of attachment quality and conduct, peer, and emotional problems. Results revealed partial support for the hypothesized mediation effect: the schema domain of disconnection/rejection acted as a mediator in the links between insecure attachment and peer problems and emotional problems. Further analysis of these effects revealed that different types of maladaptive schemas were involved in both types of psychopathology. Altogether, findings suggest that treatment of adolescent psychological problems may need to target the improvement of attachment relationships with peers and parents and the correction of underlying cognitive schemas.

## Introduction

Epidemiological research has indicated that a substantial minority of youths show clear signs of psychopathology at some point during their childhood (Costello et al. 2003). The most frequently diagnosed disorders can be divided into two broad categories, namely emotional disorders such as anxiety disorders and depression, and behavioral disorders such as attention-deficit and disruptive behavior disorders (see also Ford et al. 2003). Current models on the etiology of mental health problems in young people rely on the valuable insights of developmental psychopathology, which assumes that emotional and behavioral problems in children and adolescents arise as a result of multiple vulnerability and risk factors (Wenar and Kerig 2000). One prominent risk factor that has received an increasing amount of research attention over the past decades is attachment insecurity (Cassidy and Shaver 2008). Originating from a psychodynamic tradition, it was Bowlby (1969, 1973) who assumed that due to insensitive and unresponsive caregiving in the early years of development, problems arise in the child’s ability to make strong affectional bonds to others, which then form the basis for various types of psychopathology (see Bowlby 1977). Indeed there is accumulating evidence indicating that an insecure attachment status is associated with high symptom levels of emotional as well as behavioral disorders in youths (Brumariu and Kerns 2010; Colonnesi et al. 2011; Fearon et al. 2010).

The pathogenic effect of attachment insecurity was initially explained by suggesting that the adverse early experiences with the primary caregivers (in most cases the parents) are stored in dysfunctional internal working models, which undermine proximity seeking to other persons thereby hindering an important mechanism for regulating distressing emotions and enhancing the risk for developing psychopathology (Bowlby 1969). According to Beck’s (1976, 2005) cognitive theory of psychopathology, dysfunctional internal working models can best be seen as maladaptive schemas. These schemas are formed early during children’s life as a result of negative experiences with parents and peers, and are assumed to strongly guide the aberrant cognition, emotion, and behavior as seen in many types of psychopathology (Young 1994). Thus, a theoretical model can be hypothesized in which early maladaptive schemas act as a cognitive mediator in the relation between attachment insecurity and psychopathology.

As yet, the empirical evidence for this model is fairly sparse. There is some support for the idea that attachment insecurity is linked to early maladaptive schemas. More precisely, in a 15-year longitudinal study, Simard et al. (2011) assessed attachment status in children at age 6 using a separation-reunion procedure and maladaptive schemas at age 21 by means of a self-report questionnaire. The results revealed more signs of early maladaptive schemas among the young adults that had been classified as insecurely attached when they were a child as compared to their securely attached peers (see also Mason et al. 2005). Further, clear evidence has emerged indicating that early maladaptive schemas are associated with various types of psychopathology, and this also appears to be true in youth populations (Muris 2006; Van Vlierberghe et al. 2009, 2010).

Two recent studies have examined whether early maladaptive schemas indeed mediate the relation between attachment insecurity and psychopathology. In the first study by Bosmans et al. (2010), 289 late adolescents (with a mean age of 21 years) completed a set of questionnaires for measuring attachment anxiety and avoidance (as indicators of attachment insecurity), early maladaptive schemas, and psychopathological symptoms. Non-parametric tests of mediation effects indicated that the maladaptive schema domains of disconnection/rejection (i.e., schemas referring to expectations that one’s basic needs in close relationships will not be met in a predictable manner) and other-directedness (i.e., schemas that are concerned with the excessive focus on the desires of others at the expense of one’s own needs) fully mediated the relation between attachment anxiety and psychopathology. Further, it was found that the schema domain of disconnection/rejection also partly mediated the link between attachment avoidance and psychopathology.

A similar approach was adopted by Roelofs et al. (2011) who assessed the quality of attachment relationship to parents and peers, maladaptive schemas, and symptoms of depression in 222 adolescents aged 12–18 years. In this study, lack of trust in parents and alienation from peers were the indicators of attachment insecurity, which were found to be associated with depression. Again the schema domains of disconnection/rejection (in particular the specific schemas of mistrust/abuse and social isolation) and other-directedness (in particular the schema of self-sacrifice) emerged as the cognitive mediators. Altogether, the results of both studies clearly indicate that in particular schemas regarding expectations to be disconnected and rejected mediate the relation between attachment insecurity and psychopathology.

The current study extends on the research of Bosmans et al. (2010), Roelofs et al. (2011) and examined the mediating role of early maladaptive schemas in the relationship between attachment insecurity and psychopathology. The study focused on young people aged 12–18 years because adolescence is in many ways a taxing developmental stage that may easily provoke psychopathology in vulnerable and at-risk youths (Wenar and Kerig 2000). Whereas the earlier research by Bosmans et al. (2010), Roelofs et al. (2011) made use of non-clinical participants who were recruited at the university or secondary schools, the present investigation relied on a clinical sample of youths who were referred to an outpatient treatment center. Further, the previous studies were focused on the prediction of psychopathological symptoms in general (Bosmans et al. 2010) and symptoms of depression (Roelofs et al. 2011). So thus far little is known about the specificity of early maladaptive schemas acting as a mediator in the relation between attachment insecurity and various types of psychopathological problems in youths. Cognitive theory would suggest that the pathogenic cognitive basis of emotional and behavioral disorders would be quite dissimilar (Beck 1976), and there is indeed some evidence indicating that different early maladaptive schemas are involved in various types of emotional and behavioral problems in youths (Muris 2006; Van Vlierberghe et al. 2010). With this in mind, the present study explored whether different early maladaptive schemas are found as mediators in the links between attachment insecurity on the one hand, and emotional, conduct, and peer problems on the other hand.

## Method

### Participants and Procedure

Eighty-two adolescent patients (46 boys and 36 girls) aged between 12 and 18 years were recruited at an outpatient treatment center in Maastricht, The Netherlands. During the intake process, the psychologist, psychiatrist, or social worker provided the children and parent(s) with information about the study and asked them to participate. Upon agreement adolescents completed a set of questionnaires (see below). As shown in Table 1, the vast majority of the adolescents had at least one DSM-IV-TR Axis 1 diagnosis (97.6 %), with attention-deficit/hyperactivity, relational problems, and pervasive developmental disorders being most prevalent. More than half of the adolescents also had a comorbid Axis 1 disorder (69.5 %) and a substantial minority displayed clear signs of an Axis 2 disorder (19.5 %). Between 28 and 48.8 % exhibited scores in the clinical range on the *Strengths and Difficulties Questionnaire* (SDQ), a well-known index of psychopathology in youths (see also below), which further underlines the clinical nature of the sample. Most adolescents were Caucasian and a global estimation based on the educational level/unemployment status of the parents suggests that between 19.5 and 41.5 % has a lower socio-economic background. About one-fourth of the adolescents (26.8 %) came from broken families.

**Table 1:** Descriptive characteristics of the clinically referred adolescents and their families

	*M* (SD) or *N* (%)
*Adolescents*	
Age	14.21 (1.67)
Gender (boys/girls)	46/36 (56.1/43.9)
DSM-IV-TR primary diagnosis Axis 1	80 (97.6)
Attention-deficit/hyperactivity disorder	20 (24.4)
Adjustment disorders	6 (7.3)
Anxiety disorders	6 (7.3)
Depressive disorders	4 (4.9)
Disorder of adolescence NOS	1 (1.2)
Identity problem	6 (7.3)
Oppositional defiant/conduct disorder	4 (4.9)
Pervasive developmental disorders	10 (12.2)
Learning disorders	6 (7.3)
Relational problems	16 (19.5)
Somatoform disorder	1 (1.2)
No clinical diagnosis	2 (2.4)
At least 1 comorbid DSM-IV-TR Axis 1 diagnosis	57 (69.5)
DSM-IV-TR primary diagnosis Axis 2	16 (19.5)
SDQ scores in clinical range^a^	
Total problems	33 (40.2)
Emotional problems	23 (28.0)
Conduct problems	25 (30.5)
Hyperactivity problems	40 (48.8)
Peer problems	26 (31.7)
Prosocial behavior (deficit)	32 (39.0)
Families*	
Age mother	44.46 (5.60)
Age father	46.79 (5.22)
Ethnicity (non-Caucasian)	4 (4.9)
Education mother (low)	27 (32.9)
Education father (low)	34 (41.5)
Working status mother (unemployed)	25 (30.5)
Working status father (unemployed)	16 (19.5)
Family status (% broken)	22 (26.8)

DSM-IV-TR = Diagnostic and statistical manual of mental disorders, fourth edition—text revision. *NOS* Not otherwise specified. *SDQ* Strengths and Difficulties Questionnaire (self-report version). ^a^Scoring > 1 SD above the normative mean (Meltzer et al. 2000). *Some family background variables were estimated because of missing data

### Questionnaires

The *Inventory of Parent and Peer Attachment* (IPPA; Armsden and Greenberg 1987) consists of two scales that are scored independently: the parent scale (28 items) and the peer scale (25 items). Each scale contains items that are related to three domains of attachment quality: trust items reflect the degree of mutual understanding and respect (e.g., “My parents/friends respect my feelings”), communication items assess the extent of spoken communication (e.g., “I tell my parents/friends about my problems and troubles”), while alienation items tap feelings of anger and interpersonal isolation (e.g., “My parents/friends don’t understand what I am going through these days”). Items have to be rated on 5-point Likert scales with 1 = *almost never true*, 2 = *seldom true*, 3 = *sometimes true*, 4 = *often true*, and 5 = *almost always true*. IPPA parent and peer scales scores can be obtained by summing across relevant items. Previous research has shown that the IPPA is reliable in terms of internal consistency (e.g., Armsden and Greenberg 1987, 1989), and there is also support for its validity as demonstrated by meaningful links with other instruments for assessing attachment (Muris et al. 2001) and parental bonding (Gullone and Robinson 2005).

An adolescent version of the 75-item version of the *Young Schema Questionnaire* (Young and Brown 1990; YSQ-A, see Van Vlierberghe et al. 2010) was employed to assess 15 core maladaptive schemas (Young 1994; Young et al. 2003). Items [e.g., “During my childhood, nobody supported me when I was sad or scared” (emotional deprivation), “I am a failure” (failure to achieve), and “I have the feeling that the world is a dangerous place” (vulnerability to harm and illness)] are scored on a 6-point rating scale ranging from 1 = completely untrue for me to 6 = describes me perfectly. There are five items per schema, and each schema score can be calculated by summing the ratings on these five items. In addition, factor analytic research of the YSQ-A has indicated that individual schemas load on five higher-order schema domains, namely disconnection and rejection (i.e., mistrust/abuse, emotional deprivation, defectiveness/shame, social isolation/alienation, abandonment, and emotional inhibition), impaired autonomy (i.e., dependency/incompetence, vulnerability to harm/illness, enmeshment/undeveloped self, failure to achieve), impaired limits (i.e., entitlement/grandiosity, insufficient self-control/discipline), other-directedness (i.e., subjugation, self-sacrifice), and overvigilance/inhibition (i.e., emotional inhibition, unrelenting standards (Van Vlierberghe et al. 2010). So far, little is known about the psychometric properties of the YSQ-A, but available research has indicated that the reliability (internal consistency) of schema and domain scales are satisfactory (Roelofs et al. 2011; Van Vlierberghe et al. 2010).

The *SDQ* consists of 25 items describing positive and negative attributes of children and adolescents that can be allocated to 5 subscales of 5 items each: emotional symptoms, conduct problems, hyperactivity-inattention, peer problems, and prosocial behavior. Each item has to be scored on a 3-point scale with 0 = ‘not true’, 1 = ‘somewhat true’, and 2 = ‘certainly true’. Subscale scores can be computed by summing scores on relevant items (after recoding reversed items; range 0–10). In the present study, we focused on emotional problems, conduct problems and peer problems as attachment insecurity and early maladaptive schemas can be considered as relevant factors involved in the etiology of these types of psychopathology. Previous research by Goodman (1999, 2001) has shown that the SDQ possesses good psychometric properties. More specifically, the internal consistency of various SDQ subscales is moderate but acceptable given the shortness of various scales. Furthermore, correlations among parent, teacher, and self-report SDQ scores compare favorably to cross-informant correlations as obtained with other psychopathology measures. Finally, evidence has been obtained for the validity of the SDQ. That is, SDQ scores were found to correlate in a theoretical meaningful way with other measures of psychopathology [e.g., Achenbach’s (1991) *Child Behavior Checklist*; Muris et al. 2003; Muris et al. 2004].

### Data Analysis

The Statistical Package of Social Sciences (SPSS) was used for computing descriptive statistics, internal consistency coefficients, and correlations among the main study variables. A stepwise procedure was employed to investigate the hypothesized model attachment insecurity → maladaptive schemas → psychopathological symptoms. First, regression analyses were conducted to identify which parent and peer attachment quality variable was most clearly associated with various symptoms scores in this sample of clinically referred adolescents. Second, a bootstrapping procedure (with *N* = 5,000 bootstrap re-samples) was carried out (as multiple mediators were involved; Preacher and Hayes 2008) to investigate which maladaptive schema domains mediated the link between the identified attachment quality variable and various types of symptoms. In this analysis, we always controlled for the influence of the two other attachment quality scales. Third, in case a schema domain emerged as a significant mediator, the mediating effects of individual maladaptive schemas comprising that domain were explored by means of a further bootstrapping analysis. To assess indirect effects in a bootstrapping procedure, 95 % percentile confidence intervals (CIs) for the parameter estimates of various mediating variables were calculated. A parameter estimate was considered significant in case zero did not fall in the CI.

## Results

### Reliability of Questionnaires

Reliability coefficients of the IPPA trust and communication scales were good, but those obtained for the alienation scales were insufficient (Cronbach’s α’s being .48 and .42 for the parent and peer scales, respectively). The reliability of the YSQ-A maladaptive schema domains was good, with Cronbach’s alphas in the .73–.93 range, and this appeared also true for most individual schema scales (Cronbach’s α’s between .60 and .90, with the only exception being the enmeshment/undeveloped self scale, which displayed an alpha of .45). For the SDQ emotional problems subscale, a satisfactory alpha coefficient of .79 was found. For the conduct and peer problems subscales, fairly low alphas of .56 and .49 were obtained.

### Correlations among Attachment, Schema Domains, and Problems

Pearson correlations among the main study variables are shown in Table 2. The main results of this analysis can be summarized as follows. First, some IPPA attachment scales were substantially correlated. That is, communication was positively associated with trust (parent: *r* = .75, *p* < .001; peer: *r* = .72, *p* < .001) but negatively related to alienation (parent: *r* = −.45, *p* < .001; peer: *r* = −.36, *p* < .001), whereas trust was negatively linked to alienation (only parent: *r* = −.40, *p* < .001). However, correlations among the parent and peer scales of the IPPA were generally low. For example, when looking at corresponding subscales, only for alienation a significant cross-informant correlation was observed (*r* = .27, *p* < .05). This implies that these clinically referred adolescents rated the attachment relations to parents as quite different from the attachment relationships to peers. Second, with regard to the relations between attachment and maladaptive schemas, it was found that IPPA peer subscales were more convincingly connected to YSQ-A schema domains than IPPA parent subscales. Note further that trust and communication were negatively related to schema domain scores, whereas positive correlations were observed between alienation and schema domain scores. Third, correlations among YSQ-A scales were between .36 and .73 (all *p*’s < .001), indicating that maladaptive schema domains to some extent share common variance. Fourth, significant correlations were also observed between attachment quality as measured by the IPPA and symptoms scores as indexed by the SDQ. The most robust correlations were observed between trust in parents and conduct problems (*r* = −.56, *p* < .001), trust in peers and peer problems (*r* = −.52, *p* < .001), communication with peers and peer problems (*r* = −.41, *p* < .001), and alienation from peers and emotional problems (*r* = .40, *p* < .001). Fifth, schema domain scores were positively related to emotional symptoms (*r*’s between .38 and .58, *p*’s < .001) and, although to a lesser extent, peer problems (*r*’s between .23 and .42, *p*’s < .05/.001). Impaired limits was the only schema domain that was significantly associated with conduct problems (*r* = .36, *p* < .001). Sixth and finally, SDQ symptoms scores were not strongly related. The only significant correlation was observed between emotional and peer problems (*r* = .28, *p* < .05).

**Table 2:** Correlations among self-report questionnaires measuring attachment quality, schema domains, and psychopathological symptoms

	(1)	(2)	(3)	(4)	(5)	(6)	(7)	(8)	(9)	(10)	(11)	(12)	(13)
IPPA													
(1) Parent–trust													
(2) Parent–communication	.75**												
(3) Parent–alienation	−.45**	−.40**											
(4) Peer–trust	.13	.22*	−.19										
(5) Peer–communication	−.05	.09	.01	.72**									
(6) Peer–alienation	−.35**	−.17	.27*	−.36**	−.18								
YSQ-A													
(7) Disconnection/rejection	−.32*	−.27*	.42**	−.60**	−.34*	.48**							
(8) Impaired autonomy	−.18	−.13	.13	−.50**	−.44**	.37**	.73**						
(9) Impaired limits	−.21	−.09	.02	−.10	−.22	.15	.36**	.53**					
(10) Other-directedness	−.18	−.13	.30*	−.34*	−.04	.31*	.57**	.56**	.43**				
(11) Overvigilance/inhibition	−.14	−.14	.26*	−.28*	−.28*	.35**	.44**	.49**	.38**	.48**			
SDQ													
(12) Emotional problems	−.19	−.07	.22*	−.26*	−.08	.40**	.58**	.56**	.38**	.43**	.43**		
(13) Conduct problems	−.56**	−.33*	.22*	.15	.17	.06	.10	.09	.36**	.15	.08	.13	
(14) Peer problems	−.08	−.25*	.14	−.52**	−.41**	.30*	.42**	.23*	.00	.26*	.32*	.28*	.01

*N* = 82. *IPPA* Inventory of Parent and Peer Attachment, *YSQ-A* Young Schema Questionnaire for Adolescents, *SDQ* Strengths and Difficulties Questionnaire. *p < .05, **p < .001

### Mediation Effects of Schemas in the Relation Between Attachment and Symptoms

**Fig. 1:**
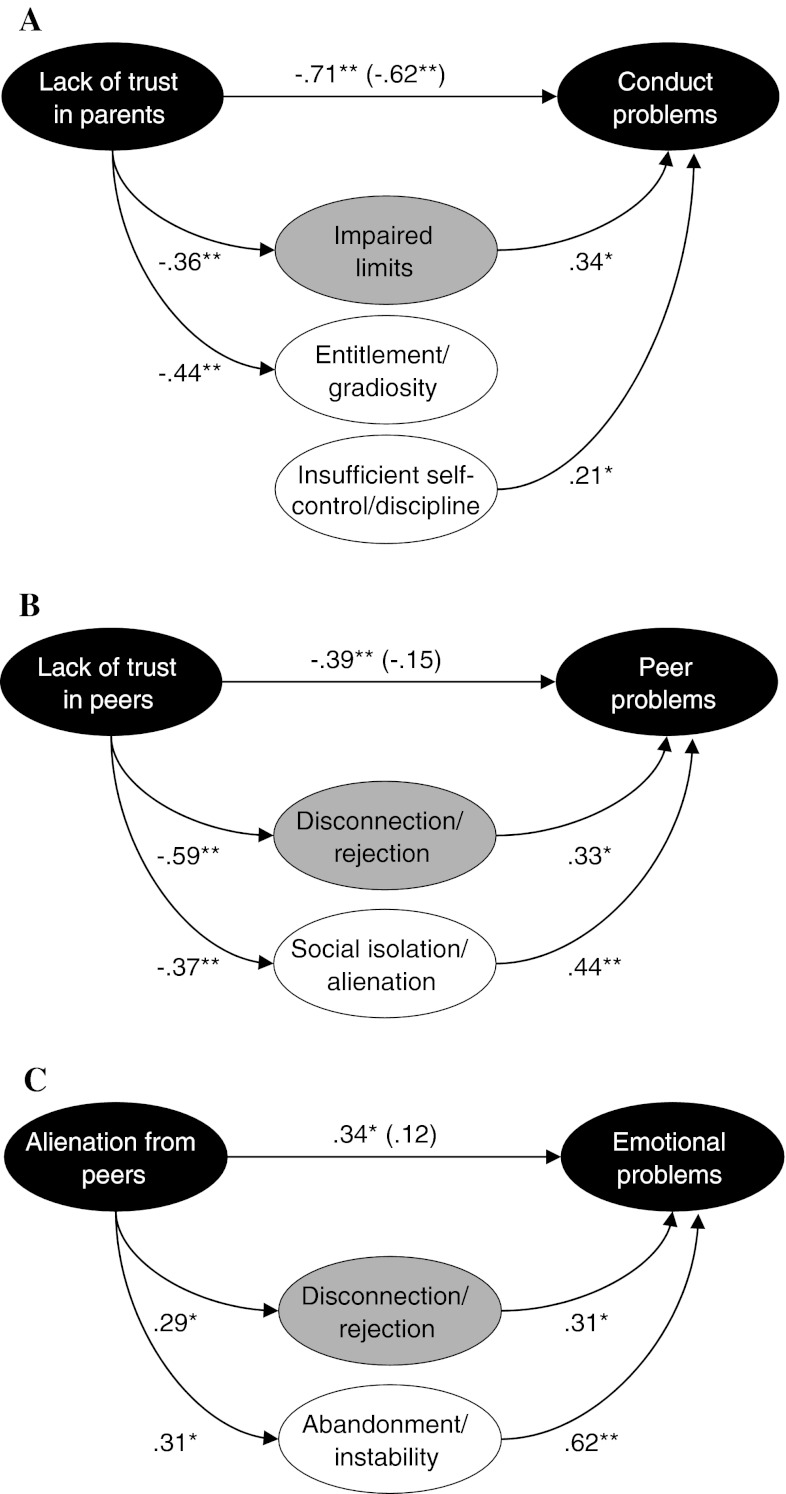
Results of the bootstrapping procedure investigating schema domains (*grey ovals*) and individual maladaptive schemas (*white ovals*) as mediators in the relations between attachment insecurity and psychopathological symptoms *Note*. Standardized β-values are shown. *p < .05, **p < .001. In all cases, we controlled for the effects of the two non-included attachment scales

Regression analyses were performed to identify which attachment quality variable was most clearly associated with various symptoms scores. The analyses using IPPA parent scales revealed no significant predictor of emotional problems. However, trust in parents emerged as a significant unique predictor of conduct problems (β = −.71, *t* = −4.92, *p* < .001), whereas communication with parents made a unique contribution to peer problems (β = −.42, *t* = −2.56, *p* < .05). Bootstrapping analyses indicated that 42 % of the variance in conduct problems was explained by a model in which the link between lack of trust in parents and conduct problems was partly mediated by the domain of impaired limits [95 % CI (−.33 to −.00]. However, further investigation of which individual schemas within the domain of impaired limits were responsible for the observed indirect effect, revealed that none of the schemas really acted as a mediator. Instead, significant links were found between lack of trust in parents and entitlement/grandiosity, and between insufficient self-control/discipline and conduct problems (see Fig. 1a).

The bootstrapping analysis examining the mediating role of schema domains in the relationship between communication with parents and peer problems yielded no support for a mediation model. Here none of the paths between the predictor and the mediating schema domains attained significance, which means that a basic requirement for the hypothesized mediation model was not met.

A regression analysis employing IPPA peers scales revealed no significant independent peer attachment predictor in the case of conduct problems. However, a regression analyses did identify lack of trust in peers as a unique predictor of peer problems (β = −.39, *t* = −2.65, *p* < .05), and alienation from peers as a significant predictor of emotional problems (β = .34, *t* = 3.03, *p* < .01). Bootstrapping analysis seemed to suggest that the domain of disconnection/rejection acted as a mediator in the relationship between lack of trust in peers and peer problems, with 38 % of the variance being explained by this model. The relation between lack of trust in peers and peer problems became non-significant when the mediating schema domain was added to the model. However, inspection of the statistics revealed that zero just fell in the 95 % CI (−.44 to .00), which means that formally the criterion for a mediation effect was not met. Yet, when studying this effect in more detail by investigating the role of individual schemas, results clearly indicated that social isolation/alienation acted as a significant mediator in the link between lack of trust in peers and peer problems [95 % CI (−.41 to −.03)] (see Fig. 1b).

The bootstrapping analysis examining which schema domains acted as an mediator in the relationship between alienation from peers and emotional symptoms, found support for a mediating effect of disconnection/rejection [95 % CI (.01–.27)]. The link between this attachment variable and emotional symptoms was no longer significant when the indirect path via disconnection/rejection was included in the model. In total the model accounted for 40 % of the variance in emotional problems. An additional analysis showed that the individual schema of abandonment/instability [95 % CI (.06–.39)] mainly accounted for the observed mediation effect between alienation from peers and emotional symptoms (see Fig. 1c).

## Discussion

The current study investigated the relationships between attachment insecurity, maladaptive schemas, and various types of psychopathological symptoms in a sample of clinically referred adolescents, suffering from a variety of DSM-IV Axis 1 and 2 disorders. A mediation model was hypothesized in which schema domains and maladaptive schemas acted as mediators in the relations between indices of attachment quality and conduct, peer, and emotional problems. On a global level, the results of the present study can be summarized as follows. First, evidence was obtained showing that the links between attachment insecurity and psychopathological symptoms were indeed (to some extent) mediated by early maladaptive schemas (see also Bosmans et al. 2010; Roelofs et al. 2011). Second, support was also found for the idea that various types of problems were associated with different maladaptive schemas. This is of course in keeping with Beck’s (1976, 2005) content-specificity hypothesis, which assumes that psychological disorders can be differentiated on the basis of their underlying cognitions.

A more detailed look at the present findings revealed that the schema domain disconnection/rejection acted as a mediator in the relation between alienation from peers as an index of attachment insecurity on the one hand and emotional problems on the other hand. This result is partly in keeping with that obtained by Roelofs et al. (2011) who also observed that disconnection/rejection is involved in depression symptoms of adolescents, and corroborates previous research in adults indicating that people with affective problems generally expect that their needs for security and safety will not be met in a predictable manner (e.g., Calvete et al. 2005). Further analysis revealed that in particular the schema of abandonment/instability carried this mediation effect. While this finding is in disagreement with Roelofs et al. (2011) who documented the schemas of mistrust/abuse and social isolation/alienation as significant mediators in the link between attachment insecurity and depression symptoms, it should be noted that in various other studies on anxiety and depression in adults patient populations abandonment/instability did emerge as an important correlate of such emotional symptoms (Glaser et al. 2002; Petrocelli et al. 2001; Stopa et al. 2001; Welburn et al. 2002). Thus differences in the type of population (non-clinical versus clinical) may account for the somewhat diverging results.

Indications were found that the relationship between attachment insecurity and peer problems was also mediated by the schema domain disconnection/rejection. Here the maladaptive schema of social isolation/alienation played a significant role. So far, nothing has been reported in the literature on the association between maladaptive schemas and this type of problems in adolescents. However, the result that social isolation/alienation appears to be involved in peer problems is barely surprising as this specific maladaptive schema is concerned with feeling isolated and different from other people and having the idea that one is no part of any group or community (Young 1994). Note that especially during adolescence it is important for young people to acquire their position in the social network of peers (Wenar and Kerig 2000), and so it is easy to see that such maladaptive underlying cognitive structure is inconvenient to achieve this goal.

On first sight the schema domain of impaired limits seemed to mediate the relationship between lack of trust in parents and conduct problems. However, a closer examination of this effect indicated that none of the schemas belonging to this domain really acted as a mediator. Instead it was found that the schema of insufficient self-control/discipline made a unique contribution to conduct problems, which is in keeping with previous findings showing that this schema is involved in anger (Calvete et al. 2005) and aggression (Tremblay and Dozois 2009). As the present sample included quite a number of adolescents with ADHD and ODD, it makes sense that their conduct problems were at least in part based on difficulties to exercise sufficient self-control and low frustration tolerance.

Various types of attachment insecurity were involved in the models explaining the three types of psychopathological symptoms. More precisely, alienation from peers was associated with emotional problems, lack of trust in parents with conduct problems, and lack of trust in peers with peer problems. Although the three IPPA scales display considerable correlations, factor analytic research has generally indicated that they represent three discernable factors (e.g., Pace et al. 2011). This suggests that the IPPA scales tap different aspects of insecure attachment, some of which play a unique role in various types of adolescent psychopathology. Further, it should be noted that the cross-informant correlations of the three IPPA scales were rather small, which means that adolescents make a differentiation between attachment relationships to parents and those to peers (see also Armsden and Greenberg 1987, 1989). The results showed that a parent scale (lack of trust) emerged as a unique predictor of conduct problems, whereas peer scales (alienation and lack of trust) were found to be independent predictors of emotional and peer problems. Thus, in terms of insecure attachment relationships, parents may be more important for our understanding of adolescents’ conduct problems, while peers may be more relevant for emotional and peer problems.

A number of limitations of this study need to be highlighted. First, it should be borne in mind that the present investigation relied on a cross-sectional data set. Although the testing of the theoretical model was clearly grounded in the existing literature, it is obvious that no conclusions on cause-effect relations among the assessed variables can be drawn. Second, the study merely relied on adolescents’ self-report. Although this is certainly a defendable method for assessing internal phenomena such as cognitive schemas, it is also clear that other constructs (e.g., conduct problems) may be better measured via the parents. In the end, a multi-method approach (i.e., assessing all variables in adolescents as well as parents) would have been preferable as this would have enabled us to cross-validate the current findings and to reduce the problem of shared-method variance. Third, for several reasons one might question the use of the SDQ for measuring psychopathological symptoms. To begin with, some subscales (i.e., conduct problems, peer problems) displayed insufficient internal consistency. This has also been observed in previous studies (Muris et al. 2004; Van Widenfelt et al. 2003), and probably can be ascribed to the fact that these scales only contain a limited set of items of which some are reversely scored. In the meantime, research has demonstrated that such low alpha coefficients do not devaluate the validity of these SDQ scales (Goodman 2001). In addition, although we made an attempt to investigate the links between insecure attachment, maladaptive schemas, and various types of psychopathological symptoms, it can be argued that the SDQ still does not differentiates a number of important problems in adolescents. For example, the emotional problems scale combines symptoms of anxiety and depression, although recent studies have indicated that different maladaptive schemas seem to be involved in both types of problems (e.g., Cámara and Calvete 2012). Fourth and finally, we only only investigated attachment insecurity and early maladaptive schemas as antecedents of psychopathology. Of course, this is a simplification of reality as many more variables are involved in the etiology of various problems (e.g., parental rearing, stressful events, genetics; Wenar and Kerig 2000), which is also illustrated by the fact that our models only accounted for approximately 40 % of the variance in adolescents’ problems scores.

A strong point of this study was that it relied on a sample of clinically referred adolescents, and in spite of the aforementioned shortcomings, the findings may still have implications for the treatment of this population. To begin with, the findings suggest that it may be helpful to target the intervention on an amelioration of the relationships with parents and peers. Attachment-based and peer-mediated interventions (e.g., social skills training) could be implemented to repair relational ruptures and rebuild trustworthy relationships with parents and peers. In addition, in current practice, adolescents’ problems are increasingly tackled with cognitive-behavioral therapy (Barrett and Ollendick 2004). Most of the available treatment programs aim at restructuring negative thinking in daily situations into more positive thinking. The present findings suggest that problems in adolescents are partly based on underlying maladaptive schemas, and so the effects of treatment could be optimized by also targeting these deeply rooted pathogenic cognitions (Schmeck 2008).
